# Supplementary material for: Improvement of PR8-Derived Recombinant Clade 2.3.4.4c H5N6 Vaccine Strains by Optimization of Internal Genes and H103Y Mutation of Hemagglutinin
Source: Vaccines (Basel). 2020 Dec 20;8(4):781. doi: 10.3390/vaccines8040781 (PMC7766170; doi:10.3390/vaccines8040781)
Supplement: Supplementary file 1 [file vaccines-08-00781-s001.pdf]

Supplementary Table 1. Amino acid identities of used NP, M and NS1 genes to those of wild-type HPAIVs 2018-2020 isolates

Sequence Identity (%)	NP				M1			
	H5N1 (*n* = 21)	H5N8 (*n* = 26)	H5N6 (*n* = 59)	Challenge virus[a]	H5N1 (*n* = 21)	H5N8 (*n* = 26)	H5N6 (*n* = 59)	Challenge virus
PR8	93.14	93.98	93.59	92.80	91.72	92.69	92.01	92.80
SNU50-5	98.40	99.40	98.93	98.30	93.95	96.11	95.13	95.70
01310	97.73	97.95	97.76	97.40	93.86	95.93	94.96	94.90
0028	98.06	98.55	98.33	98.10	94.19	94.13	93.93	94.90

[a] Challenge virus used in chicken experiment that was wild-type clade 2.3.4.4c H5N6 virus (A/Mandarin_duck/Korea/K16-187-3/2016)

Supplementary Table 2. Comparison of amino acid sequences of M2e.

		H5N1<br>(*n* = 21)	H5N8<br>(*n* = 26)	H5N6<br>(*n* = 37)	**SNU50-5<br>(H5N1)**	**01310<br>(H9N2)**	0028<br>(H9N2)	PR8
M2e	MSLLTEVET<br>PTRNEWECR<br>CSDSSD	.........<br>.........<br>......[a]	.........<br>....G....<br>N.....	.........<br>....G....<br>......	.........<br>....G....<br>K.....	.........<br>....G....<br>KY...E	.........<br>...DG....<br>K.N..N	.........<br>.I.....G.<br>..NG..[b]
		.........<br>H........<br>......	.........<br>....G....<br>K.....	.........<br>....G....<br>K.....				

[a] same amino acid with the peptide sequence was denoted with dot

[b] M2e of PR8 had potential N-glycosylation site (from position 21 to 23, NGS)

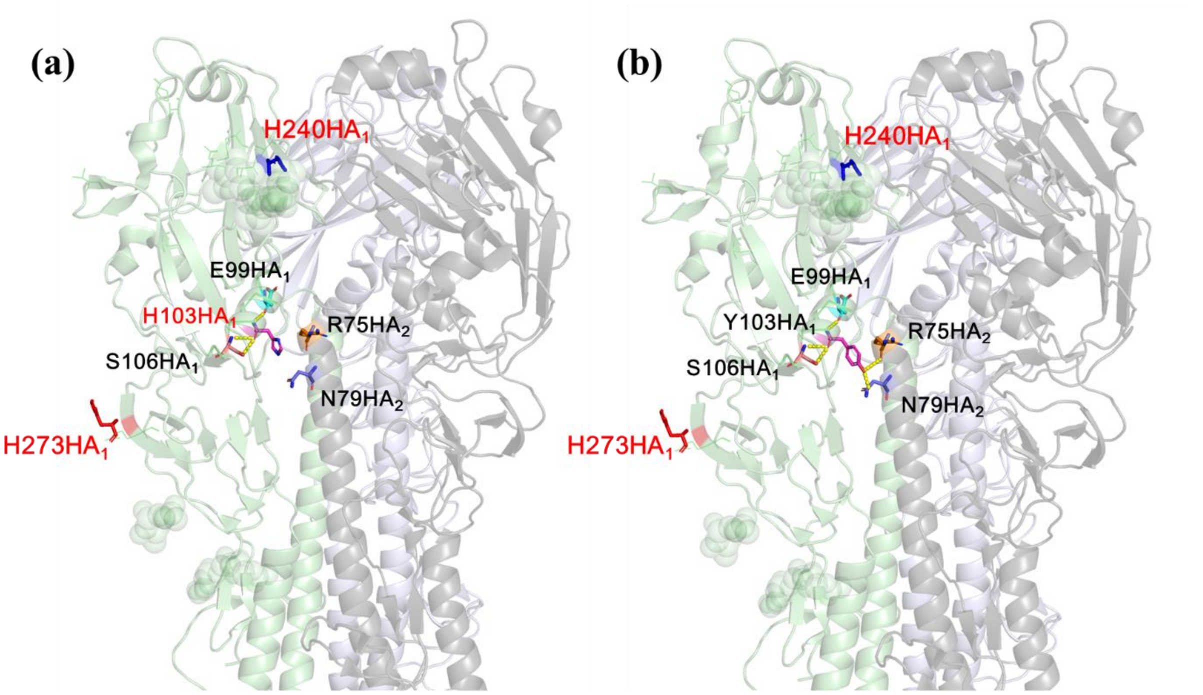

Supplementary Figure 1. Three-dimensional structure of clade 2.3.4.4c H5N6 HA trimer. HA trimer structure was reorganized 5hu8 PDB file with the Pymol Molecular Graphics System. Each HA monomer was differently colored and inter-/intra-molecular interaction of residue 103 of HA1 (103HA$_1$) with other residue was depicted as yellow line. Clade 2.3.4.4c H5N6 have two more histidine (H240HA$_1$ and H273HA$_1$) than A/Indonesia/5/2005(H5N1). (a) when clade 2.3.4.4c H5 have H103HA$_1$, it does not interact with other HA monomer. (b) However, H103HA$_1$ is mutatted into tyrosine (Y103HA$_1$), it acquired polar contact with HA$_2$ of other monomer (R75HA$_2$, N79HA$_2$).

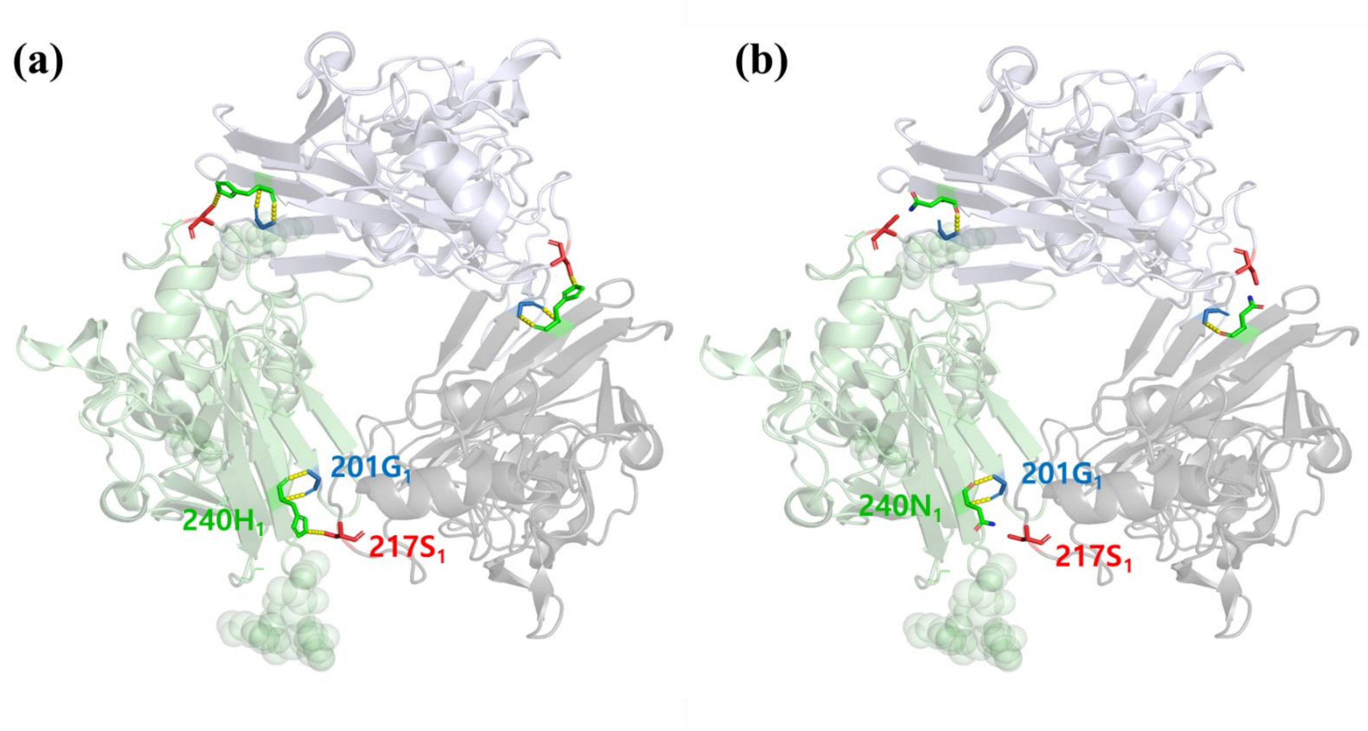

Supplementary Figure 2. Structure and interaction at the globular head domain of HA trimer. Globular head of HA trimer of clade 2.3.4.4c H5N6 virus was constructed using 5hu8 PDB file and the Pymol Molecular Graphics System. Interaction between HA monomers was showed in top view. (a) clade 2.3.4.4c H5N6 had histidine at position 240 of HA1 and $240H_1$ formed inter-molecular hydrogen bonds with $201G_1$ and intra-molecular hydrogen bond with $217S_1$ in other HA monomer. (b) But, other H5N1 viruses had asparagine at position 240 and it didn’t interact with $217S_1$ in other monomer.
